# Association of *LIPC *-250G>A polymorphism and several environmental factors with serum lipid levels in the Guangxi Bai Ku Yao and Han populations

**DOI:** 10.1186/1476-511X-9-28

**Published:** 2010-03-11

**Authors:** Li Meng, Yin Ruixing, Li Yiyang, Long Xingjiang, Li Kela, Liu Wanying, Zhang Lin, Lin Weixiong, Yang Dezhai, Pan Shangling

**Affiliations:** 1 Department of Cardiology, Institute of Cardiovascular Diseases, the First Affiliated Hospital, 22 Shuangyong Road, Nanning 530021, Guangxi, People's Republic of China; 2 Department of Molecular Biology, Medical Scientific Research Center, 22 Shuangyong Road, Nanning 530021, Guangxi, People's Republic of China; 3 Department of Pathophysiology, School of Premedical Sciences, Guangxi Medical University, 22 Shuangyong Road, Nanning 530021, Guangxi, People's Republic of China

## Abstract

**Background:**

The association between -250G>A polymorphism in the promoter region of the hepatic lipase gene (*LIPC*) and plasma high-density lipoprotein cholesterol (HDL-C) concentration is contradictory in diverse ethnics. Bai Ku Yao is an isolated subgroup of the Yao minority in China. This study was designed to detect the association of *LIPC *-250G>A (rs2070895) polymorphism and several environmental factors with serum lipid levels in the Guangxi Bai Ku Yao and Han populations.

**Methods:**

A total of 778 subjects of Bai Ku Yao and 648 participants of Han Chinese aged 15-80 were randomly selected from our previous stratified randomized cluster samples. Genotyping of the *LIPC *-250G>A was performed by polymerse chain reaction and restriction fragment length polymorphism combined with gel electrophoresis, and then confirmed by direct sequencing.

**Results:**

The levels of serum total cholesterol (TC), HDL-C, low-density lipoprotein cholesterol (LDL-C) and apolipoprotein (Apo) AI were lower in Bai Ku Yao than in Han (*P *< 0.01 for all). The frequencies of GG, GA and AA genotypes were 50.0%, 43.3% and 6.7% in Bai Ku Yao, and 35.7%, 50.6% and 13.7% in Han (*P *< 0.01); respectively. The frequencies of G and A alleles were 71.7% and 28.3% in Bai Ku Yao, and 61.0% and 39.0% in Han (*P *< 0.01). The levels of HDL-C and the ratio of ApoAI to ApoB in Bai Ku Yao were lower in GG genotype than in GA or AA genotype (*P *< 0.05-0.01). The levels of TC, HDL-C, LDL-C and ApoB in Han were lower in GG genotype than in GA or AA genotype (*P *< 0.05-0.01). The levels of HDL-C and the ratio of ApoAI to ApoB in Bai Ku Yao, and the levels of HDL-C, LDL-C and ApoB in Han were correlated with genotype and/or allele (*P *< 0.05 for all). Serum lipid parameters were also correlated with age, sex, alcohol consumption, cigarette smoking, blood pressure, body weight, and body mass index in both ethnic groups.

**Conclusions:**

The differences in the serum lipid profiles between the two ethnic groups might partly result from different genotypic frequency of *LIPC *-250G>A or different *LIPC*-enviromental interactions.

## Introduction

Coronary artery disease is the most common cause of morbidity and mortality in the industrialized nations and is of growing concern in developing countries. Epidemiological studies have shown that dyslipidaemia, including high levels of plasma or serum total cholesterol (TC) [1,2], triglycerides (TGs) [3,4], low-density lipoprotein cholesterol (LDL-C) [5,6] and apolipoprotein B (ApoB) [7,8], and low levels of high-density lipoprotein cholesterol (HDL-C) [9,10], is a risk factor for the progression of atherosclerosis and the development of coronary artery disease. It is well known that plasma lipid concentrations are modulated by both environmental factors such as demographics [11], diet [12], alcohol consumption [13], cigarette smoking [13,14], obesity [15], exercise [16], hypertension [17] as well as genetic factors. Family and twin studies have shown that genetic polymorphism could account for 40-80% of the interindividual variation in plasma lipid concentrations [18-20].

Hepatic lipase is an enzyme that regulates the metabolism of low-density lipoprotein (LDL), intermediate-density lipoprotein (IDL), and HDL particles [21,22]. High hepatic lipase activity is associated with low HDL-C level, and hepatic lipase converts large, TG-rich HDL_2_-C into small, dense HDL_3_-C [23]. Hepatic lipase also catalyzes the hydrolysis of TG and phospholipids in large, buoyant LDL to form more atherogenic small, dense LDL particles [24,25]. Hepatic lipase activity is determined by gender, visceral obesity, insulin resistance, diet, and genotype. Approximately 20-30% of the individual variation in hepatic lipase activity is due to the presence of a common polymorphism in the promoter region of the hepatic lipase gene [26]. The human hepatic lipase gene (*LIPC*), located on chromosome 15q21, is comprised of nine exons and eight introns, spans over 30 kb of DNA, and encodes a protein of 449 amino acids [27,28]. *LIPC *variants have been found to affect lipase activity. Four different promoter polymorphisms of the *LIPC *have been identified (-250G>A, -514C>T, -710T>C, and -763A>G), which are in complete linkage disequilibrium [29,30]. The -250G>A substitution in the promoter region of the *LIPC *has been found to be associated with modifications of plasma lipid concentrations [31-38] and the risk of coronary artery disease [39,40] in some studies but not in others [41-44].

There are fifty-six ethnic groups in China. Han is the largest group and Yao is the eleventh largest minority among the 55 minority groups according to the population size. Bai Ku Yao (White-trouser Yao), an isolated subgroup of the Yao minority, is named so because all the men wear white knee-length knickerbockers. The population size is about 30000. Because of isolation from the other ethnic groups, the special customs and cultures including their clothing, intra-ethnic marriages, dietary habits, and corn wine and rum intakes are still completely preserved to the present day. In a previous epidemiologic study, we found that the serum lipid concentrations were lower in Bai Ku Yao than in Han Chinese from the same region [45]. This ethnic difference in serum lipid concentrations is still not well known. We hypothesized that there may be significant differences in some genetic polymorphisms between the two ethnic groups. Therefore, the aim of the present study was to detect the association of *LIPC *-250G>A (rs2070895) polymorphism and several environmental factors with serum lipid levels in the Guangxi Bai Ku Yao and Han populations.

## Materials and methods

### Study population

A total of 778 subjects of Bai Ku Yao who reside in Lihu and Baxu villages in Nandan County, Guangxi Zhuang Autonomous Region, People's Republic of China were randomly selected from our previous stratified randomized cluster samples [45]. The ages of the subjects ranged from 15 to 80 years, with an average age of 40.28 ± 13.78 years. There were 405 males (52.1%) and 373 females (47.9%). All subjects were peasants. The subjects accounted for 2.59% of total Bai Ku Yao population. During the same period, a total of 648 people of Han Chinese who reside in the same villages were also randomly selected from our previous stratified randomized cluster samples [45]. The mean age of the subjects was 40.22 ± 15.52 years (range 15 to 80). There were 315 men (48.6%) and 333 women (51.4%). All of them were also peasants. All study subjects were essentially healthy and had no evidence of any chronic illness, including hepatic, renal, or thyroid. The participants with a history of heart attack or myocardial infarction, stroke, congestive heart failure, diabetes or fasting blood glucose ≥ 7.0 mmol/L determined by glucose meter were also rejected. The participants were not taking medications known to affect serum lipid levels (lipid-lowering drugs such as statins or fibrates, beta-blockers, diuretics, or hormones). The present study was approved by the Ethics Committee of the First Affiliated Hospital, Guangxi Medical University. Informed consent was obtained from all subjects after they received a full explanation of the study.

### Epidemiological survey

The survey was carried out using internationally standardized methods, following a common protocol [46]. Information on demographics, socioeconomic status, and lifestyle factors was collected with standardized questionnaires. Overall physical activity was ascertained with the use of a modified version of the Harvard Alumni Physical Activity Questionnaire [47], which included questions about the number of hours per day (mean of a regular weekday and a regular weekend day) spent sleeping and in sedentary, light, moderate, and vigorous activities; the interviewer ensured that the total time added up to 24 h. The alcohol information included questions about the number of liangs (about 50 g) of rice wine, corn wine, rum, beer, or liquor consumed during the preceding 12 months. Alcohol consumption was categorized into groups of grams of alcohol per day: ≤ 25 and ≥ 25. Smoking status was categorized into groups of cigarettes per day: ≤ 20 and ≥ 20. At the physical examination, several anthropometric parameters, such as height, weight, and waist circumference were measured. Sitting blood pressure was measured three times with the use of a mercury sphygmomanometer after an at least 15-min rest, and the average of the three measurements was used for the level of blood pressure. Systolic blood pressure was determined by the first Korotkoff sound, and diastolic blood pressure by the fifth Korotkoff sound. Body weight, to the nearest 50 grams, was measured using a portable balance scale. Subjects were weighed without shoes and in a minimum of clothing. Height was measured, to the nearest 0.5 cm, using a portable steel measuring device. From these two measurements body mass index (BMI, kg/m^2^) was calculated. Waist circumference was measured with a nonstretchable measuring tape, at the level of the smallest area of the waist, to the nearest 0.1 cm.

### Biochemical analysis

A venous blood sample of 8 ml was obtained from all subjects between 8 and 11 AM, after at least 12 hours of fasting, from a forearm vein after venous occlusion for few seconds in a sitting position. 3 ml was collected into glass tubes and allowed to clot at room temperature, and used to determine serum lipid levels, and the remaining 5 ml was transferred to tubes with anticoagulate solution (4.80 g/L citric acid, 14.70 g/L glucose, and 13.20 g/L tri-sodium citrate) and used to extract DNA. Immediately following clotting serum was separated by centrifugation for 15 minutes at 3000 rpm. The levels of TC, TG, HDL-C, and LDL-C in samples were determined by enzymatic methods with commercially available kits, Tcho-1, TG-LH (RANDOX Laboratories Ltd., Ardmore, Diamond Road, Crumlin Co. Antrim, United Kingdom, BT29 4QY), Cholestest N HDL, and Cholestest LDL (Daiichi Pure Chemicals Co., Ltd., Tokyo, Japan), respectively. Serum ApoAI and ApoB levels were detected by the immunoturbidimetric immunoassay using a commercial kit (RANDOX Laboratories Ltd.). All determinations were performed with an autoanalyzer (Type 7170A; Hitachi Ltd., Tokyo, Japan) in the Clinical Science Experiment Center of the First Affiliated Hospital, Guangxi Medical University [45,48].

### DNA amplification and genotyping

Genomic DNA was isolated from peripheral blood leukocytes using the phenol-chloroform method [17]. The extracted DNA was stored at 4°C until analysis. Genotyping of the *LIPC *-250G>A was performed by polymerase chain reaction and restriction fragment length polymorphism (PCR-RFLP). PCR amplification was performed using 5'-GGCAAGGGCATCTTTGCTTC-3' and 5'-GGTCGATTTACAGAAGTGCTTC-3' (Sangon, Shanghai, China) as the forward and reverse primer pairs, respectively. Each amplification reaction was performed in a total volume of 25 mL, containing 10 × PCR buffer (1.8 mM MgCl_2_) 2.5 μL, 1 U Taq polymerase, 2.5 mmol/L of each dNTP (Tiangen, Beijing, China) 2.0 μL, 20 pmol/L of each primer and 50 ng of genomic DNA, processing started with 94°C for 5 min and 30 cycles at 94°C for 30 s, 58.8°C for 30 s and 72°C for 30 s. This was followed by a final extension at 72°C for 4 min. Then 10 U of *Dra*I enzyme was added directly to the PCR products (10 μL) and digested at 37°C overnight. 3 μL of the digestive products were loaded on 12% polyacrylamide ready gels and then were separated by electrophoresis for 25 min at 300 mV. The gel was stained with silver nitrate cream (0.15%) for 15 min, and then added 100 ml of NaOH (1.5%) and 2.2 ml of formaldehyde after pour off the silver nitrate cream, jolted slowly untill the gel was visualized. The digestive products can be directly observed. Genotypes were scored by an experienced reader blinded to epidemiological and lipid results. Six samples (GG, GA and AA genotypes in two, respectively) detected by the PCR-RFLP were also confirmed by direct sequencing. The PCR product was purified by low melting point gel electrophoresis and phenol extraction, and then the DNA sequence were analyzed in Shanghai Sangon Biological Engineering Technology & Services Co., Ltd., China.

### Diagnostic criteria

The normal values of serum TC, TG, HDL-C, LDL-C, ApoAI, ApoB, and the ratio of ApoAI to ApoB in our Clinical Science Experiment Center were 3.10-5.17, 0.56-1.70, 0.91-1.81, 2.70-3.20 mmol/L, 1.00-1.78, 0.63-1.14 g/L, and 1.00-2.50, respectively. The individuals with TC > 5.17 mmol/L and/or TG > 1.70 mmol/L were defined as hyperlipidemic [45,48]. Hypertension was diagnosed according to the criteria of 1999 World Health Organization-International Society of Hypertension Guidelines for the management of hypertension [49,50]. The diagnostic criteria of overweight and obesity were according to the Cooperative Meta-analysis Group of China Obesity Task Force. Normal weight, overweight and obesity were defined as a BMI < 24, 24-28, and >28 kg/m^2^, respectively [45,48-50].

### Statistical analyses

Epidemiological data were recorded on a pre-designed form and managed with Excel software. All statistical analyses were done with the statistical software package SPSS 11.5 (SPSS Inc., Chicago, Illinois). Quantitative variables were expressed as mean ± standard deviation (SD), and qualitative variables as percentages. Allele frequency was determined via direct counting, and the standard goodness-of-fit test was used to test the Hardy-Weinberg equilibrium. Differences in genotype distribution between the groups were obtained using the chi-square test. The difference in general characteristics between Bai Ku Yao and Han was tested by the Student's unpaired *t*-test. The association of genotypes with lipid variables was tested by analysis of covariance (ANCOVA). Sex, age, BMI, blood pressure, alcohol intake, cigarette smoking were adjusted for the statistical analysis. In order to evaluate the association of serum lipid levels with genotypes and several environment factors, multivariate logistic regression analysis was also performed. A *P *value of less than 0.05 was considered statistically significant.

## Results

### General characteristics and serum lipid levels

Table 1 gives the general characteristics between the Bai Ku Yao and Han populations. The levels of systolic blood pressure, pulse pressure, serum TC, HDL-C, LDL-C, ApoAI and the ratio of ApoAI to ApoB were lower in Bai Ku Yao than in Han (*P *< 0.01 for all), whereas physical activity, BMI and the percentages of subjects who smoked cigarettes were higher in Bai Ku Yao than in Han (*P *< 0.01 for each). There were no significant differences in diastolic blood pressure, TG, ApoB levels, age structure, the percentages of subjects who consumed alcohol, or the ratio of male to female between the two ethnic groups (*P *> 0.05).

**Table 1:** The general characteristics and serum lipid levels between the Bai Ku Yao and Han populations

Parameter	Bai Ku Yao	Han Chinese	*t*(χ^2^)	*P*
Number	778	648		
Male/female	405/373	315/333	-1.519	0.129
Age (years)	40.28 ± 13.78	40.22 ± 15.52	-1.850	0.065
Body mass index (kg/m^2^)	22.31 ± 2.86	21.69 ± 2.84	4.166	0.000
Physical activity (h/week)	46.71 ± 12.78	43.56 ± 11.96	4.771	0.000
Systolic blood pressure (mmHg)	116.30 ± 15.62	119 ± 17.55	-3.757	0.000
Diastolic blood pressure (mmHg)	74.66 ± 9.17	74.82 ± 10.80	-0.286	0.775
Pulse pressure (mmHg)	41.63 ± 11.54	44.81 ± 12.19	-5.028	0.000
Cigarette smoking [n(%)]				
Nonsmoker	526(67.6)	518(79.9)		
≤20 cigarettes/day	136(17.5)	30(4.6)		
>20 cigarettes/day	116(14.9)	100(15.4)	57.560	0.000
Alcohol consumption [n(%)]				
Nondrinker	487(62.6)	398(61.4)		
≤25 g/day	197(25.3)	174(26.9)		
>25 g/day	94(12.1)	76(11.7)	0.434	0.805
Total cholesterol (mmol/L)	4.27 ± 0.92	4.63 ± 1.00	-7.010	0.000
Triglycerides (mmol/L)	1.28 ± 1.11	1.25 ± 0.98	0.531	0.596
HDL-C (mmol/L)	1.64 ± 0.42	1.79 ± 0.50	-6.292	0.000
LDL-C (mmol/L)	2.51 ± 0.75	2.72 ± 0.80	-5.147	0.000
Apolipoprotein (Apo) AI (g/L)	1.29 ± 0.33	1.40 ± 0.36	-6.092	0.000
ApoB (g/L)	0.83 ± 0.22	0.82 ± 0.21	0.541	0.588
ApoAI/ApoB	1.68 ± 0.79	1.79 ± 0.62	-2.814	0.005

HDL-C, highdensity lipoprotein cholesterol; LDL-C, low-density lipoprotein cholesterol

### Results of electrophoresis and genotyping

**Figure 1:**
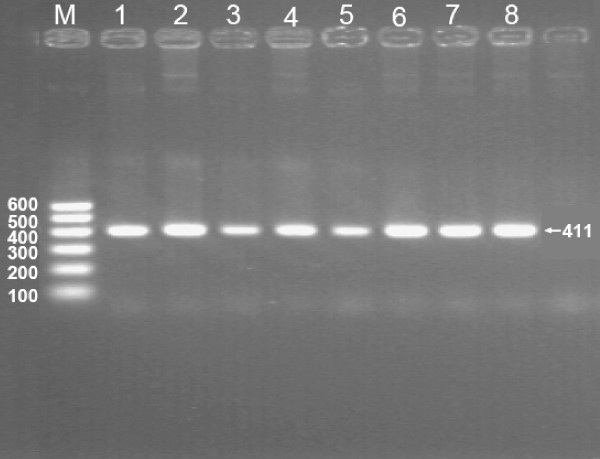
**Electrophoresis of PCR products of the samples**. Lane M, 100-bp marker ladder; lanes 1--8, samples. The 411-bp bands are the target genes.

**Figure 2:**
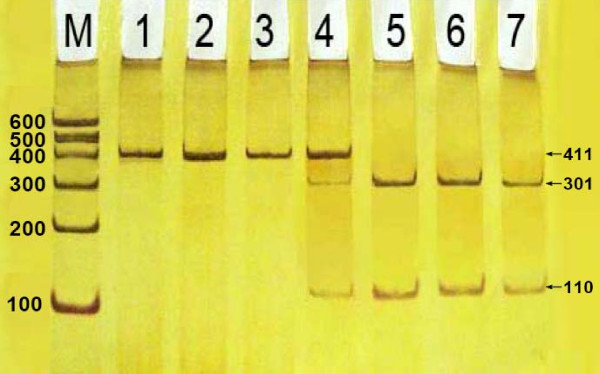
**Genotyping of PCR products of the samples**. Lane M, 100 bp Marker Ladder; Lanes 1-3, GG genotype (411 bp); Lane 4, GA genotype (411 bp, 301 bp and 110 bp); and lanes 5-7, AA genotype (301 bp and 110 bp)

After the genomic DNA of the samples was amplified by PCR and imaged by 2% agarose gel electrophoresis, the purpose gene of 411-bp nucleotide sequences could be found in all samples (Figure 1). The genotypes identified were named according to the presence or absence of the enzyme restriction sites, when a G to A transversion at nucleotide position 250 of the *LIPC*. The presence of the cutting site indicates the -250A allele, while its absence indicates the -250G allele. Thus, the GG genotype is homozygote for the absence of the site (band at 411 bp), GA genotype is heterozygote for the presence and absence of the site (bands at 411-, 301- and 110-bp), and AA genotype is homozygote for the presence of the site (bands at 301- and 110- bp; Figure 2).

### Genotypic and allelic frequencies

The frequencies of the *LIPC *-250G>A alleles and genotypes are shown in Table 2. The frequencies of G and A alleles were 71.7% and 28.3% in Bai Ku Yao, and 61.0% and 39.0% in Han (*P *< 0.01). The frequencies of GG, GA and AA genotypes were 50.0%, 43.3% and 6.7% in Bai Ku Yao, and 35.7%, 50.6% and 13.7% in Han (*P *< 0.01); respectively.

**Table 2:** Genotypic and allelic frequencies between the Bai Ku Yao and Han populations [n(%)]

Group		Genotype			Allele	
			
	n	GG	GA	AA	G	A
Bai Ku Yao	778	389 (50.0)	337 (43.3)	52 (6.7)	115(71.7)	441(28.3)
Han Chinese	648	231(35.7)	328 (50.6)	89 (13.7)	790(61.0)	506(39.0)
χ^2^	-	38.565			36.598	
*P*	-	0.000			0.000	

### Results of sequencing

**Figure 3:**
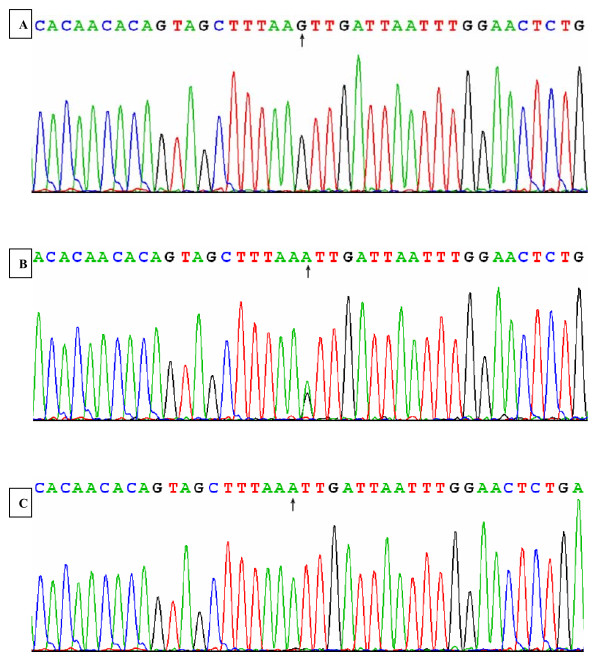
**A part of the nucleotide sequence in the *LIPC *--250G>A locus**. (A) GG genotype; (B) GA genotype; (C) AA genotype

The results shown as GG, GA and AA genotypes by PCR-RFLP, GG, GA and AA genotypes were also confirmed by sequencing (Figure 3).

### Genotypes and serum lipid levels

As shown in Table 3, the levels of HDL-C and the ratio of ApoAI to ApoB were significant differences among the three genotypes in the Bai Ku Yao population (*P *< 0.01 for each). There were also significant differences in the levels of TC, HDL-C, LDL-C and ApoB among the three genotypes in the Han population (*P *< 0.05-0.001) after adjustment for all the covariates. There were no significant differences in the levels of TG and ApoAI among the three genotypes in the both ethnic groups (*P *> 0.05).

**Table 3:** The *LIPC *--250G>A genotypes and serum lipid levels between the Bai Ku Yao and Han populations

Group	Genotype	n	TC (mmol/L)	TG (mmol/L)	HDL-C (mmol/L)	LDL-C (mmol/L)	ApoAI (g/L)	ApoB (g/L)	ApoAI/ApoB
Bai Ku Yao	GG	389	4.23 ± 0.79	1.26 ± 1.11	1.59 ± 0.42	2.48 ± 0.64	1.28 ± 0.32	0.82 ± 0.21	1.62 ± 0.72
	GA	337	4.32 ± 1.06	1.25 ± 1.09	1.67 ± .41^b^	2.56 ± 0.86	1.27 ± 0.33	0.84 ± 0.23	1.70 ± 0.81
	AA	52	4.27 ± 0.86	1.58 ± 1.22	1.75 ± 0.44^a^	2.36 ± 0.71	1.40 ± 0.38	0.79 ± 0.23	1.98 ± 1.02^bc^
*F*	-	-	0.380	1.371	5.427	1.464	1.982	1.526	5.073
*P*	-	-	0.684	0.254	0.005	0.232	0.139	0.218	0.007
Han Chinese	GG	231	4.39 ± 0.79	1.20 ± 0.80	1.77 ± 0.50	2.52 ± 0.56	1.40 ± 0.35	0.76 ± 0.16	1.76 ± 0.62
	GA	328	4.65 ± 1.06^b^	1.17 ± 1.20	1.82 ± 0.51	2.73 ± 0.86^b^	1.40 ± 0.36	0.83 ± 0.22^b^	1.79 ± 0.64
	AA	89	4.69 ± 0.96^a^	1.30 ± 1.02	1.93 ± 0.48^a^	2.77 ± 0.79^a^	1.39 ± 0.36	0.84 ± 0.21^b^	1.87 ± 0.51
*F*	-	-	5.901	1.017	3.267	6.304	1.800	9.834	1.023
*P*	-	-	0.003	0.362	0.039	0.002	0.166	0.001	0.360

TC, total cholesterol; TG, triglycerides; HDL-C, high-density lipoprotein cholesterol; LDL-C, low-density lipoprotein cholesterol; ApoAI, apolipoprotein AI; ApoB, apolipoprotein B; ApoAI/ApoB, the ratio of apolipoprotein AI to apolipoprotein B; ^*a*^*P *< 0.05 and ^*b*^*P *< 0.01 in comparison with GG genotype of same ethnic group; ^*c*^*P *< 0.05 in comparison with GA genotype of same ethnic group

### Risk factors for the lipid parameters

Multivariate logistic regression analysis showed that the levels of HDL-C and the ratio of ApoAI to ApoB were correlated with genotype in the Bai Ku Yao population (*P *< 0.01 for each), whereas the levels of HDL-C, LDL-C and ApoB were correlated with genotype and/or allele in the Han population (*P *< 0.05-0.01). The lipid parameters were also found to correlate with gender, age, BMI, alcohol consumption, cigarette smoking, blood pressure, and physical activity in the both ethnic groups (Table 4).

**Table 4:** Correlative factors for the lipid paramerers between the Bai Ku Yao and Han populations

Group	Lipid	Relative factor	Regression coefficient	Standard error	*t*	*P*
Bai Ku Yao	TC	Age	0.026	0.007	12.193	0.000
	TG	Gender	-1.357	0.293	21.494	0.000
		Body mass index	0.268	0.080	11.084	0.001
	HDL-C	Age	0.028	0.006	18.742	0.000
		Alcohol consumption	0.492	0.110	19.828	0.000
		Genotype	0.029	0.009	9.629	0.002
		Systolic blood pressure	-0.013	0.006	5.312	0.021
	LDL-C	Age	0.021	0.007	8.440	0.004
	ApoAI	Age	0.028	0.010	8.562	0.003
		Alcohol consumption	1.014	0.176	33.347	0.000
	ApoAI/ApoB	Cigarette smoking	-0.412	0.200	4.237	0.040
		Alcohol consumption	1.372	0.204	45.362	0.000
		Genotype	0.223	0.067	11.256	0.001
Han Chinese	TC	Age	0.026	0.006	16.564	0.000
		Pulse pressure	-0.025	0.009	7.525	0.006
	TG	Cigarette smoking	0.712	0.143	24.861	0.000
		Physical activity	-0.412	0.161	6.558	0.010
	HDL-C	Gender	0.666	0.237	7.925	0.005
		Age	0.014	0.006	5.674	0.017
		Cigarette smoking	-0.515	0.163	9.955	0.002
		Physical activity	0.786	0.145	29.495	0.000
		Body mass index	-0.081	0.029	7.995	0.005
		Genotype	0.412	0.210	3.899	0.048
	LDL-C	Age	0.025	0.007	14.006	0.000
		Alcohol consumption	-0.685	0.201	11.556	0.001
		Pulse pressure	-0.026	0.009	7.486	0.006
	ApoAI	Gender	0.656	0.291	5.069	0.024
		Alcohol consumption	0.762	0.208	13.461	0.000
		Physical activity	1.143	0.293	15.193	0.000
		Systolic blood pressure	0.016	0.006	5.946	0.015
	ApoB	Age	0.024	0.010	5.961	0.015
		Genotype	0.665	0.245	7.383	0.007
		Gender	1.773	0.486	13.303	0.000
		Physical activity	0.509	0.256	3.965	0.046
		Diastolic blood pressure	0.035	0.015	5.492	0.019
		Allele	-2.062	0.670	9.474	0.002
	ApoAI/ApoB	Gender	1.773	0.486	13.303	0.000
		Alcohol consumption	0.955	0.279	11.693	0.001
		Physical activity	0.509	0.256	3.965	0.046
		Diastolic blood pressure	0.035	0.015	5.492	0.019
		Allele	-2.062	0.670	9.474	0.002

TC, total cholesterol; TG, triglycerides; HDL-C, high-density lipoprotein cholesterol; LDL-C, low-density lipoprotein cholesterol; ApoAI, apolipoprotein AI; ApoB, apolipoprotein B; ApoAI/ApoB, the ratio of apolipoprotein AI to apolipoprotein B

## Discussion

The present study shows that the serum levels of TC, HDL-C, LDL-C, ApoAI and the ratio of ApoAI to ApoB were lower in Bai Ku Yao than in Han. There were no significant differences in serum TG and ApoB levels between the two ethnic groups. These findings are consistent with those of our previous studies in a large population [45]. It is well known that dyslipidaemia is a complex trait caused by multiple environmental and genetic factors and their interactions. Bai Ku Yao is an isolated subgroups of the Yao minority in China. Strict intra-ethnic marriages have been performed in this population from time immemorial. Therefore, we hypothesized that the hereditary characteristic and genotypes of some lipid metabolism-related genes in this population may be different from those in Han Chinese.

The frequency of the less common haplotype was found to range between 0.15 to 0.21 among Caucasians [41], 0.32 among Brazilian [39], 0.39 among Taiwanese-Chinese [33], 0.45-0.53 among African Americans [41,51] and 0.47 among Japanese-Americans [41]. The allele frequencies between white and black subjects are quite different: the less common allele of the *LIPC *polymorphisms in white subjects was the more common allele in black subjects [41,51]. In the present study, we show that the frequency of the *LIPC *-250G allele was higher in Bai Ku Yao than in Han. The frequency of GG genotype was also higher in Bai Ku Yao than in Han. The frequencies of the *LIPC *-250G allele and GG genotype in the Bai Ku Yao and Han populations were also higher than those of previous studies in Caucasians [51,52], These results indicate that the allelic variation of the *LIPC *-250G>A may have an ethnic specificity.

The potential relationship between the *LIPC *-250G>A polymorphism and plasma or serum lipid levels in humans has been evaluated in a large number of studies. However, previous findings on the association of this polymorphism with the changes in plasma lipid levels are inconsistent. Several studies have reported significant association between the *LIPC *-250G>A polymorphisms and HDL-C levels [32-35,38], whereas several reports failed to find a significant genetic effect on HDL-C concentrations [41-44]. Zambon *et al *[41] found that the promoter polymorphism significantly influenced HDL_2_-C but not HDL-C levels. In the present study, we found that the -250A carriers were associated with high HDL-C levels and the ratio of ApoAI to ApoB in Bai Ku Yao, and high TC, HDL-C, LDL-C and ApoB concentration in Han. The reason for this discrepancy between the two ethnic groups is currently unknown. In a previous studies, Lindi *et al *[34] found that the -250AA genotype was associated with high serum LDL-C concentration, and that the monounsaturated fatty acids-enriched diet reduced serum LDL-C concentration especially in subjects with the -250AA genotype. The impact of n-3 fatty acids on lipid and glucose metabolism did not differ among genotypes of the -250G>A polymorphism. Hepatic lipase also contributes to hepatic uptake of ApoB-containing lipoproteins [53]. Thus, it can be speculated that LDL catabolism may be marginally disturbed in subjects with the -250AA genotype compared to those with other genotypes and this could lead to slightly increased serum LDL-C concentrations. In another study, Deeb *et al *[54] have shown, by transient transfection assays of the murine hepatocyte cell line AML12, that the -514T allele of the *LIPC *promoter has approximately 30% lower activity than the C allele. But the -250G>A polymorphism does not seem to have any impact on promoter activity in this transient transfection system. In a metabolic ward study, Juo and colleagues [30] found that 38% of the variation of the hepatic lipase activity was associated with the *LIPC *promoter haplotypes, but only 25% of variation in HDL-C is associated with hepatic lipase activity. Thus, with many other factors affecting HDL-C levels, one would not expect to observe significant associations between HDL-C and the *LIPC *promoter haplotypes in all studies. Tahvanainen *et al *[42] have shown that carriers of the A allele have significantly higher amounts of TG in IDL, LDL, and HDL particles, making them more buoyant among subjects with lower hepatic lipase activities. This may be due to heterogeneity of the study populations or the presence of impaired glucose tolerance, which might have masked the effect of the -250G>A polymorphism on lipid and lipoprotein levels. Because it is currently unknown which of the promoter polymorphisms is functionally important and what are possible mechanisms of its action, it is possible that other polymorphism(s), which are in linkage disequilibrium with the *LIPC *promoter polymorphisms, in the same gene or/and another gene are responsible for the development of dyslipidaemia.

In addition to gene polymorphisms, a number of other factors such as demographics [11], diet [12], alcohol consumption [13], cigarette smoking [13,14], obesity [15], exercise [16], hypertension [17] modulate plasma lipid levels. These factors might partly explain why the association between HDL-C concentration and the -250G>A polymorphism was not observed in several previous studies. For example, heavy smokers have, on average, 9% lower HDL-C levels than matched nonsmokers [14]. Obesity is one of the most important factors in reducing HDL-C levels [15,55]. Evidences of association in different populations with different lifestyles and diet might suggest that the associations found are robust to a large number of genetic and environmental factors. In the present study, we found that many confounding factors affect serum lipid levels. Serum lipid parameters were correlated with age, sex, alcohol consumption, cigarette smoking, BMI, blood pressure and physical activity. These findings suggest that the environmental factors also play an important role in determing the lipid levels in these populations. Differences in the lipid levels between the two ethnic groups could be related to factors such as differences in the genetic background, dietary patterns and lifestyle factors. Although Bai Ku Yao and Han reside in the same region, there were differences in their diet and lifestyle that might account for the observed differences in serum lipid profiles. Corn was the staple food and rice, soy, buckwheat, sweet potato, and pumpkin products were the subsidiary foods in Bai Ku Yao. Approximately 90% of the beverages were corn wine and rum. The alcohol content is about 15% (v/v). They are also accustomed to drink Hempseed soup. In contrast, rice was the staple food and corn, broomcorn, potato, and taro products were the subsidiary foods in Han. About 90% of the beverage was rice wine. The content of alcohol is about 30% (v/v). The staple and subsidiary foods are more favorable for lipid profiles in the Bai Ku Yao diet than in the Han diet. Corn contains abundant dietary fiber and plant protein. Dietary fiber can decrease serum TC levels [56]. Plant protein can promote the transportation and excretion of free cholesterol. Soy protein intake is effective in reducing TC by 9.3%, LDL-C by 12.9%, and TG by 10.5% and in increasing HDL-C by 2.4% [57]. Hypocholesterolemic activity of buckwheat protein product is far stronger than that of soy protein isolate [58]. Ludvik and his colleagues [59] found that ingestion of 4 g/day caiapo (the extract of white-skinned sweet potato) for 6 weeks reduces TC and LDL-C in type 2 diabetic patients previously treated by diet alone. Studies have demonstrated that pumpkin is a useful therapy for hypercholesterolemia through reducing oxidative stress and cholesterol levels [60]. A number of experimental and clinical studies have demonstrated that the beneficial effects of Hempseed or Hempseed oil on lipid profiles include: decreasing TC, TG and LDL-C levels [60,61], inhibiting lipid peroxidation [61], reducing atherogenic index [62], and increasing HDL-C levels [61,62].

In summary, our findings show that there was significant difference in genotypic and allelic frequencies of the *LIPC*-250G>A polymorphism between the Bai Ku Yao and Han populations. The levels of HDL-C and the ratio of ApoAI to ApoB in Bai Ku Yao, and the levels of TC, HDL-C, LDL-C and ApoB in Han were influenced by genotype and/or allele. The differences in the lipid profiles between the two ethnic groups might partly result from different genotypic frequency of *LIPC *-250G>A or different *LIPC*-enviromental interactions.

## Competing interests

The authors declare that they have no competing interests.

## Authors' contributions

LM participated in the design, undertook genotyping, and drafted the manuscript.  YR conceived the study, participated in the design, carried out the epidemiological survey, collected the samples, and helped to draft the manuscript.  LY, LX, LK, LW and ZL collaborated to the genotyping.  LW, YD and PS carried out the epidemiological survey, collected the samples, and helped to carry out the genotyping.  All authors read and approved the final manuscript.
